# SWIM tool application to expression data of glioblastoma stem-like cell lines, corresponding primary tumors and conventional glioma cell lines

**DOI:** 10.1186/s12859-018-2421-x

**Published:** 2018-11-30

**Authors:** Giulia Fiscon, Federica Conte, Paola Paci

**Affiliations:** 1 Institute for Systems Analysis and Computer Science “Antonio Ruberti”, National Research Council, Via dei Taurini 19, Rome, 00185 Italy; 2 SysBio Centre for Systems Biology, Rome, Italy

**Keywords:** Bioinformatics, Cancer stem cell, Graph theory, Glioblastoma

## Abstract

**Background:**

It is well-known that glioblastoma contains self-renewing, stem-like subpopulation with the ability to sustain tumor growth. These cells – called cancer stem-like cells – share certain phenotypic characteristics with untransformed stem cells and are resistant to many conventional cancer therapies, which might explain the limitations in curing human malignancies. Thus, the identification of genes controlling the differentiation of these stem-like cells is becoming a successful therapeutic strategy, owing to the promise of novel targets for treating malignancies.

**Methods:**

Recently, we developed SWIM, a software able to unveil a small pool of genes – called switch genes – critically associated with drastic changes in cell phenotype. Here, we applied SWIM to the expression profiling of glioblastoma stem-like cells and conventional glioma cell lines, in order to identify switch genes related to stem-like phenotype.

**Results:**

SWIM identifies 171 switch genes that are all down-regulated in glioblastoma stem-like cells. This list encompasses genes like CAV1, COL5A1, COL6A3, FLNB, HMMR, ITGA3, ITGA5, MET, SDC1, THBS1, and VEGFC, involved in “ECM-receptor interaction“ and “focal adhesion” pathways. The inhibition of switch genes highly correlates with the activation of genes related to neural development and differentiation, such as the 4-core OLIG2, POU3F2, SALL2, SOX2, whose induction has been shown to be sufficient to reprogram differentiated glioblastoma into stem-like cells. Among switch genes, the transcription factor FOSL1 appears as the brightest star since: it is down-regulated in stem-like cells; it highly negatively correlates with the 4-core genes that are all up-regulated in stem-like cells; the promoter regions of the 4-core genes harbor a consensus binding motif for FOSL1.

**Conclusions:**

We suggest that the inhibition of switch genes in stem-like cells could induce the deregulation of cell communication pathways, contributing to neoplastic progression and tumor invasiveness. Conversely, their activation could restore the physiological equilibrium between cell adhesion and migration, hampering the progression of cancer. Moreover, we posit FOSL1 as promising candidate to orchestrate the differentiation of cancer stem-like cells by repressing the 4-core genes’ expression, which severely halts cancer growth and might affect the therapeutic outcome. We suggest FOSL1 as novel putative therapeutic and prognostic biomarker, worthy of further investigation.

**Electronic supplementary material:**

The online version of this article (10.1186/s12859-018-2421-x) contains supplementary material, which is available to authorized users.

## Background

Glioblastoma multiforme (GBM) is the most frequently diagnosed brain tumor in adults [1, 2] according to the World Health Organization (WHO). Recently, the unique professional research organization CBTRUS provided a comprehensive summary of the current descriptive epidemiology of primary brain and central nervous system tumors in the United States population for diagnosis years 2008-2012 [3]. From this prospective study emerges that glioblastoma accounts for 15.1% of all primary brain tumors and 46.1% of primary malignant brain tumors; it is more common in older adults especially in males (is about 1.6 times higher in males as compared to females), and is less common in children; it has the highest incidence among all malignant tumors, with 11890 cases predicted in 2015 and 12120 in 2016.

**Fig. 1:**
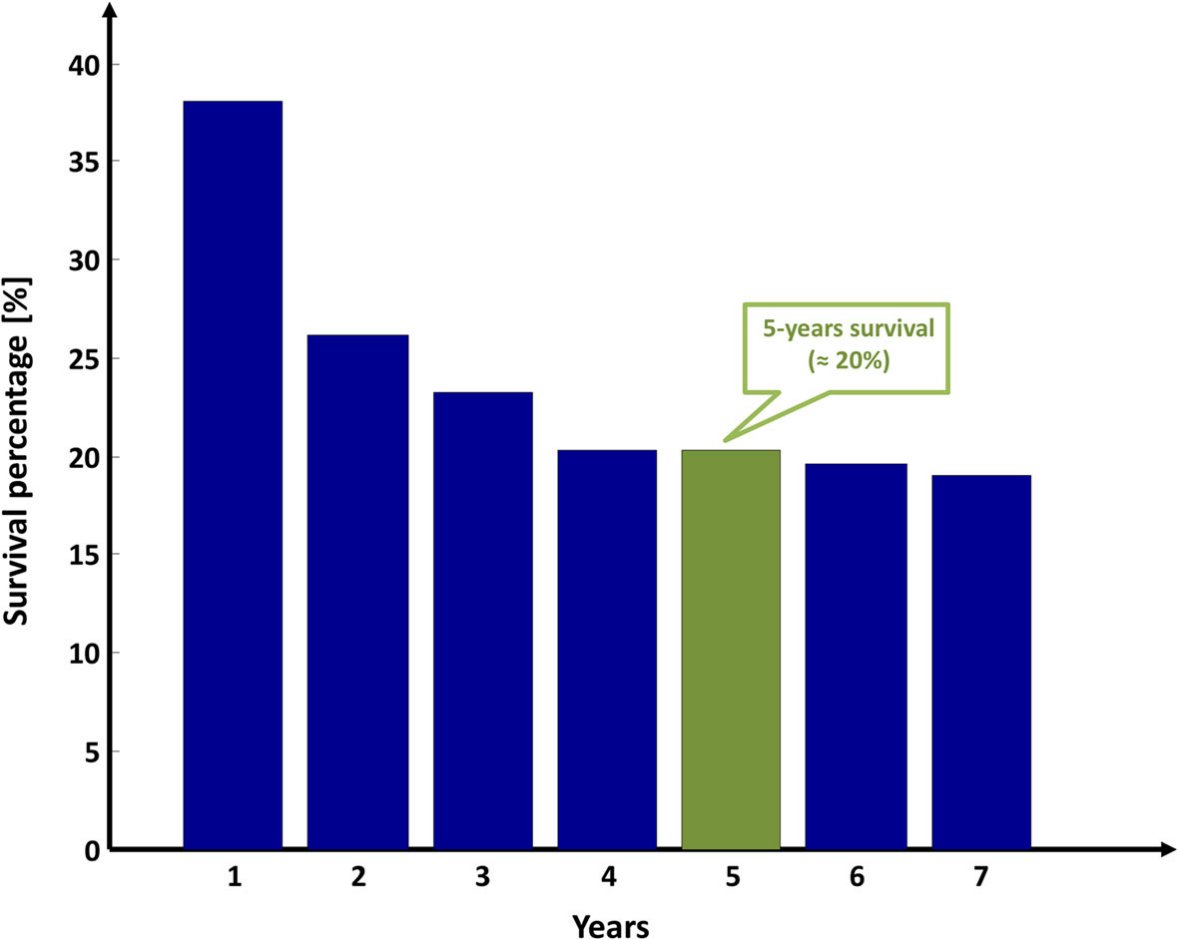
Survival percentage for GBM patients. The figure depicts the post-diagnosis survival percentage related to 161 unique GBM patients, whose clinical data were retrieved from TCGA repository in 2017 considering a follow-up of about seven years [8, 9]. The percentage of survivors at five years post-diagnosis is highlighted in green

GBM is also one of the most incurable cancer worldwide mostly due to high infiltration into the brain parenchyma making both standard therapies (e.g. radiotherapy, chemotherapy) and surgical resection generally not able to arrest the tumor development and progression [4, 5]. Despite aggressive and multimodality treatments [6, 7], GBM mortality rate remains still very high especially when compared to other cancers such as breast and lung cancer [3]. This finding is dramatically confirmed by the clinical data of 161 unique GBM patients available from The Cancer Genome Atlas (TCGA) Data Portal [8, 9]. These data point out that the 5-years survival rate is estimated to be achieved only from the 20% of the patients (Fig. 1).

Current scientific research and clinical trials have not led to a definitive cure for GBM but have contributed to both an improved understanding of the disease progression, as well as small improvements in patient outcomes to treatment. In particular, several studies identified a small percentage of the total GBM cell population that evolves along the course of the disease, forming highly heterogeneous subpopulations within the tumor mass. These cells possess radio/chemo-resistant properties and may have a role in driving tumor initiation, resistance to treatment, tumor progression, and relapse [10–16]. Due to their ability of self-renewing, proliferating, and differentiating into multiple lineages, this subpopulation of cells - known as cancer stem-like cells [17] - is held responsible for carcinogenesis not only in brain cancer [10, 13], but even in other tumors such as breast, colon, prostate, pancreatic, melanoma cancers [10, 13, 18–22]. The failure in removing these GBM cancer stem-like cells is one of the main reason behind the ineffectiveness of traditional therapies in treating glioblastoma [16]. Therefore, focusing on the characteristics of GBM stem-like cells and on necessary conditions for specific cell differentiation is a promising strategy to propose new therapeutic targets in order to improve GBM treatment efficacy and overcome drug resistance.

Here, we applied SWItchMiner SWIM [23, 24] – a software that we recently developed to unveil a small pool of genes (called switch genes) critically associated with drastic changes in cell phenotype – to the expression data obtained by Affymetrix HG-U133 Plus 2.0 microrarrays of glioblastoma stem-like (GS) cell lines, corresponding primary tumors, and conventional glioma cell lines [25], publicly available on the Gene Expression Omnibus (GEO) repository [26]. Our aim was to identify switch genes in the transition from stem-like to differentiated GBM cells.

## Methods

### Datasets

#### Schulte et al.

**Fig. 2:**
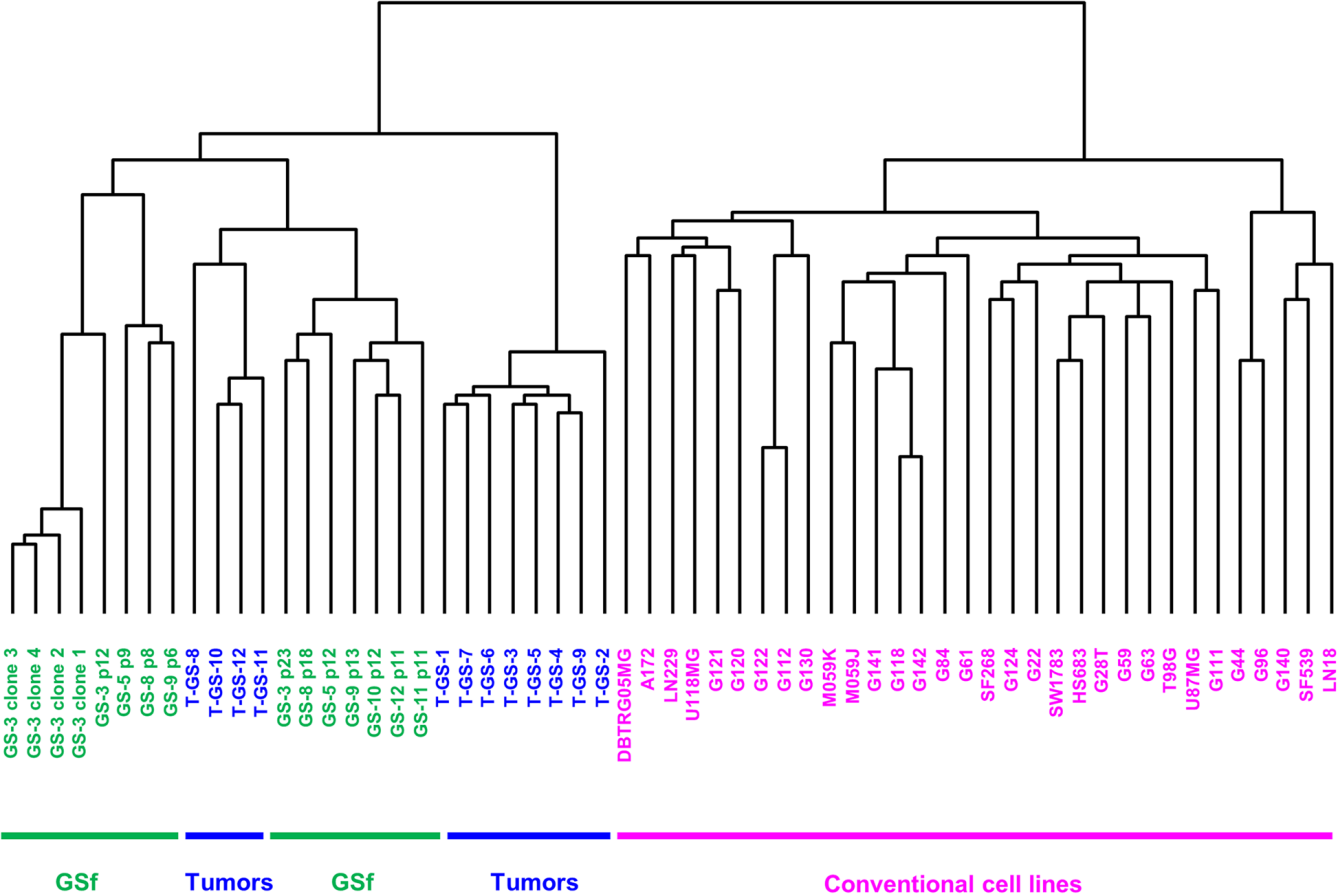
Unsupervised hierarchical cluster of tumors and cell lines. Specimens included 12 glioblastomas (blue), 15 glioblastoma full stem-like phenotype (GSf) cell lines established from these tumors (green) and 32 conventional glioma cell lines (magenta). The dendogram was obtained by using Spearman correlation as distance metrics and highlights how the GSf cell lines mirror the transcriptome of tumors more closely than conventional cell lines

The first GBM dataset analyzed for the present study is available through GEO public repository under accession number GSE23806 published on Feb 12, 2011 by [25]. Data include expression profiles of 32 conventional glioma cell lines, 12 glioblastoma stem-like (GS) cell lines, among which 7 clonal sublines derived from two GS lines, 12 original tumors, and 4 monolayer cultures established from the same tumors as GS-lines using standard serum conditions obtained by Affymetrix Human Genome U133 Plus 2.0 Array. The authors of [25] showed that only one subgroup of GS cell lines, called full stem-like phenotype (GSf), fulfilled all criteria for glioma stem cells and mirrored the transcriptome of human glioblastomas more closely than other cell lines. For this reason, in our analysis we compared the expression profiles of 23531 genes in 15 GSf cell lines and 12 original tumors with respect to 32 conventional glioblastoma cell lines (Fig. 2).

#### Verhaak et al.

The second GBM dataset analyzed for the present study is available as supplementary material of a recent study[27]. Data include microarray expression profiles of 173 core TCGA samples unified and scaled from three gene expression platforms (Affymetrix HuEx array, Affymetrix U133A array, and Agilent 244K array). The authors of this study described a robust gene expression-based molecular classification of GBM into Proneural, Neural, Classical, and Mesenchymal subtypes and integrate multidimensional genomic data to establish patterns of somatic mutations and DNA copy number. Aberrations and gene expression of EGFR, NF1, PDGFRA/IDH1, and neuron markers (e.g. NEFL, GABRA1, SYT1, SLC12A5) each define the Classical, Mesenchymal, Proneural and Neural subtypes, respectively.

#### TCGA-GBM

The third GBM dataset analyzed for the present study was downloaded from TCGA Data Portal Release 10.0 (December 2017) [8, 9]. It represents GBM normalized expression data of 161 GBM unique patients from high-throughput RNA sequencing, created by using FPKM procedure to perform the normalization (i.e. HTSeq-FPKM data). For these patients also clinical data were downloaded from TCGA in order to perform the Kaplan-Meier survival analysis.

### SWIM software

SWItchMiner (SWIM) [23] is a software with a user-friendly Graphical User Interphase (GUI) developed in MATLAB and downloadable from the supplementary materials of [23]. SWIM implements an integrated network analysis able to extract from genome-wide expression data key players (i.e. switch genes) marking the shift from one condition to another in a complex biological network. SWIM algorithm encompasses a series of well-defined steps described in the following [23].

#### Step 1: Pre-processing phase

Denoting by A and B the two conditions between which searching for switch genes and by S the total number of samples (S = samples in the condition A + samples in the condition B), this step requires the selection of two specific thresholds for removing genes whose expression across the S samples is mostly zero or change very little. The first threshold regards the maximum number of samples out of S allowed to be equal to zero. The second threshold concerns the minimum variation - measured by the Inter Quartile Range (IQR) percentile - allowed for each gene across the S samples.

#### Step 2: Filtering phase phase

This step requires the selection of two specific thresholds for removing genes whose expression between the two given conditions (A and B) does not change enough or does it without statistical significance. Considering the logarithm of the ratio between the average expression of samples in condition A and the average expression of samples in condition B (log fold-change), the first threshold allows to remove the genes falling behind, in absolute value, a fixed cutoff on the log fold-change. The second threshold concerns the smallest probability (*p*-value) for which the data allow to reject the null hypothesis (i.e. the means of the two distributions – normal and cancer - are identical) of the Student’s t-test. Actually, since this statistical test will be repeated multiple times (as many as the genes under testing), the obtained *p*-values must be adjusted. To correct multiple tests, SWIM makes use of False Discovery Rate (FDR) method [28] and thus the threshold refers to the FDR values. At end of this phase, the differentially expressed genes between conditions A and B have been identified.

#### Step 3: Building the correlation network

This step requires the selection of a threshold for building the correlation network where two nodes are connected if the absolute value of the Pearson correlation between their expression profiles exceeds a given cutoff. This threshold should reflect a right balance between the number of edges and the number of connected components of the network: the number of edges should be as small as possible in order to have a manageable network (pointing towards a higher threshold) and the number of connected components should be as small as possible in order to preserve the integrity of the network (pointing towards a smaller threshold).

#### Step 4: Finding communities in the network

To find communities in the network, SWIM makes use of the k-means algorithm [29], a method of cluster decomposition whose aim is to partition *n* objects (i.e. the nodes of the co-expression network) into *N* clusters. The goal of the clustering is expressed by an objective function that depends on the proximities of the nodes to the cluster centroids. As objective function, SWIM uses the Sum of the Squared Error (SSE), defined as follows:

$$SSE = \sum_{i=1}^{N} \sum_{x \in C_{i}} dist(c_{i},x)^{2}$$

where *N* is the number of the clusters, *C*_*i*_ is the *i*^*th*^ cluster, *x* is a node in the *i*^*th*^ cluster, *c*_*i*_ is the centroid of the *i*^*th*^ cluster. The centroid *c*_*i*_ is given by:

$$c_{i} = \frac{1}{m_{i}}\sum_{x \in C_{i}} x_{i}$$

where *m*_*i*_ is the number of nodes in the *i*^*th*^ cluster. There are as many centroids as the number of the clusters. As measurement of the proximity of two nodes, SWIM makes use of the metrics *dist*(*x*,*y*)=1−*ρ*(*x*,*y*), where *ρ*(*x*,*y*) is the Pearson correlation between expression profiles of nodes *x* and *y*. Thus, two nodes are close in the network (*dist*=0) if they are highly correlated (*ρ*=+1) on the contrary they are far apart in the network (*dist*=1) if they are highly anti-correlated (*ρ*=−1). The k-means algorithm, despite being the most widely used clustering algorithm, has some intrinsic limitations. Firstly, the number of clusters must be set in advance; secondly, it guarantees convergence only to a local minimum of SSE; thirdly, the initial position of the centroids is randomly chosen causing a dependence of the partitioning on initialization. However, some reasonable assumptions can be done and are described in the following. There is no strict method to determine the “correct” number of clusters. Among others, SWIM uses an approach - named “Scree plot” - that evaluates the behavior of the SSE function to vary the number of clusters. Then, the position of an elbow in the scree plot - i.e., where the “cliff” reaches a bottom plateau - determines an appropriate number of clusters. Since finding the global optimum of SSE is theoretically NP-hard [30], it is commonly assumed that is sufficient to carry out a number of random initialization followed by a selection of the best separated solution, measured by the lowest SSE [31]. Moreover, the partition with the lowest SSE is commonly assumed to be reproducible under repeated initializations [31]. Thus, for a given number of clusters, SWIM allows repeating the clustering many times (replicates), each with a new set of initial cluster centroid positions. For each replicate, the k-means algorithm performs iterative partitioning (iterations) until the minimum of the SSE function is reached. Then, the cluster configuration with the lowest SSE values among all replicates will be chosen, for that number of clusters.

#### Step 5: Building the heat cartography map

Once the modular structure of the complex network has been found, roles have to be assigned to each node. This is done by dividing the plan according to two parameters, the clusterphobic coefficient *K*_*π*_ and the global within-module degree *z*_*g*_. In the following, the formal definitions of these parameters for a generic node *i* [23]:

$$K_{\pi}^{i} = 1-\left(\frac{k^{in}_{i}}{k_{i}}\right)^{2}$$

$$z_{g}^{i} = \frac{k^{in}_{i}-\bar{k}_{C_{i}}}{\sigma_{C_{i}}}$$

where $k^{in}_{i}$ is the number of links of node *i* to nodes in its module *C*_*i*_, *k*_*i*_ is the total degree of node *i*, $\bar {k}_{C_{i}}$ and $\sigma _{C_{i}}$ are the average and standard deviation of the total degree distribution of the nodes in the module *C*_*i*_. This definition of *z*_*g*_ quantifies how much a node is a hub (i.e. degree exceeding 5 [32]) in its community and thus represents a measure of local connectivity. On the contrary, the parameter *K*_*π*_ evaluating the ratio of internal to external connections of a node represents a measure of global connectivity. Note that *K*_*π*_=0 when a node has only links within its module, i.e. it does not communicates with the other modules $\left (k^{in}_{i} = k_{i}\right)$. On the contrary, *K*_*π*_ is close to 1 when the majority of its links are external to its own module. The values of these two parameters define, in the plan identified by *K*_*π*_ and *z*_*g*_, a cartography made up by seven regions (R1-R7) corresponding to seven different roles of nodes in the network [33]:

1. non local hub for *z*_*g*_<2.5
  *K*_*π*_=0 Ultra-peripheral nodes (role R1)
  *K*_*π*_≤0.625 Peripheral nodes (role R2)
  0.62<*K*_*π*_≤0.8 Non-hub connectors (role R3)
  *K*_*π*_>0.8 Non-hub kinless nodes (role R4)
2. local hub for *z*_*g*_≥2.5
  *K*_*π*_=0.3 Provincial hubs (role R5)
  *K*_*π*_≤0.75 Connector hubs (role R6)
  *K*_*π*_>0.75 Kinless hubs (role R7)

Then, SWIM colors nodes in the cartography according to the Average Pearson Correlation Coefficient (APCC) between the expression profiles of each node and its nearest neighbors [32]. This representation of the network is defined as “heat cartography map”. By computing the APCC of expression over all interaction partners of each hub in protein-protein interaction (PPI) networks in yeast, the authors in [32] concluded that hubs fall into two distinct categories: date hubs that display low co-expression with their partners (low APCC) and party hubs that have high co-expression (high APCC). In the gene expression networks, the distribution of APCCs appears to be trimodal [23, 24] where, similarly to PPI networks, two peaks represent low (date hubs) and high (party hubs) positive APCC values, but with the addition of a new third peak which is characteristic of gene expression networks and represents negative APCC values. Nodes populating this peak are called “fight-club hubs”.

#### Step 6: Identification of switch genes

Looking at the heat cartography map, SWIM identifies the so-called *switch genes*: the subset of the fight-club hubs that mainly interact outside their community (role R4). In particular, they satisfy the following topological and expression features:

- being not an hub in their own cluster (*z*_*g*_<2.5);
- having many links outside their own cluster (*K*_*pi*_>0.8);
- having a negative average weight of their incident links (APCC < 0).

At the end of step 6, SWIM gives the opportunity to perform further analyses regarding the evaluation of network robustness, which is the resilience to errors, by studying the effect on the network connectivity of removing nodes by decreasing degree. In particular, SWIM evaluates the effect on the average shortest path - where the shortest path between two nodes is the minimum number of edges connecting them and the average shortest path is the mean of the shortest paths for all possible pairs of nodes in the network - of removing randomly chosen nodes, switch genes, fight-club hubs, date and party hubs. Since scale-free networks have few hubs and many non-hub nodes, they are amazingly resistant to a random removal of nodes, while the removal of hubs causes an effect known as “vulnerability to attack” to allude to the fact that the integrity of the network is destroyed.

### Functional and motif enrichment analysis

The associations between selected genes and functional annotation terms such as Gene Ontology (GO) terms [34] and KEGG pathways [35] were analyzed by using FIDEA web tool [36]. Binding motif enrichment analysis in promoter regions (identified as genomic regions spanning from -450 to +50 nucleotides with respect to transcription start sites) was performed by Pscan [37], which employs the JASPAR 2018 motif collection [38]. A *p*-value < 0.05, after adjustment for multiple testing performed with the Benjamini-Hochberg method [28], was set as threshold to identify functional annotations and regulatory motifs significantly enriched amongst the selected gene lists.

### microRNA target enrichment analysis

The predictions of microRNA (miRNA) targets and the information about the miRNA family members with their seed (i.e. positions 2 to 8 at the 5’-end of the mature miRNA sequence) were downloaded from TargetScan web site Release 7.0 (August 2015) [39]. The experimentally validated miRNA-target interactions were downloaded from miRTarBase web site Release 6.1 (September 2015) [40]. For each miRNA (selected miRNA) in the chosen database (TargetScan or miRTarBase), the hypergeometric test was used to calculate the significance of the enrichment of the list of switch genes in its targets. The relative *p*-value is computed as

$$p = 1- \sum_{i=0}^{X-1} \frac{{K \choose i}{M-K \choose N-i}}{{M \choose N}} $$

where *M* is the dimension of the universe (selected background), that is the number of all predicted (validated) miRNA-target interactions encompassed in TargetScan (miRTarBase); *K* is the number of predicted (validated) miRNA-target interactions encompassed in TargetScan (miRTarBase) for the selected miRNA; *N* is the number of switch genes (input gene list) recognized by TargetScan (miRTarBase); *X* is the number of switch genes for which predicted (validated) miRNA-target interactions, for the selected miRNA, exist.

### Network visualization and analysis

The free software package Cytoscape was used for visualizing gene correlation networks [41]. To find modules (i.e. locally dense regions) in the gene correlation network, we made use of the Cytoscape plugin MCODE [42], which weights nodes by a local neighborhood density measure and graphically displays ranked extracted modules.

### Kaplan-Meier analysis

In order to evaluate the clinical relevance of switch genes identified by SWIM, we performed Kaplan-Meier analysis [43] by using clinical and RNA-seq expression data provided by TCGA Data Portal Release 10.0 (December 2017) [8, 9], relating to 161 unique GBM patients and GBM subtype-specific patients. The patients were split into two groups (called low-expression and high-expression) according to the expression level of each switch gene. In particular, low- and high-expression groups referred to patients with expression levels lower than or greater than the 50^*th*^ percentile, respectively. For each patient cohort, the cumulative survival rates were computed according to the Kaplan-Meier method [43]. A log-rank test was performed to evaluate the *p*-value: the lower the *p*-value, the better the separation between the prognoses of the two groups. The resulting *p*-values were adjusted for multiple testing by using the Benjamini-Hochberg (FDR) procedure [28].

## Results

### Integrated network analysis of genes involved in the transition from glioblastoma stem-like to conventional cell lines reveals fight-club hubs

**Fig. 3:**
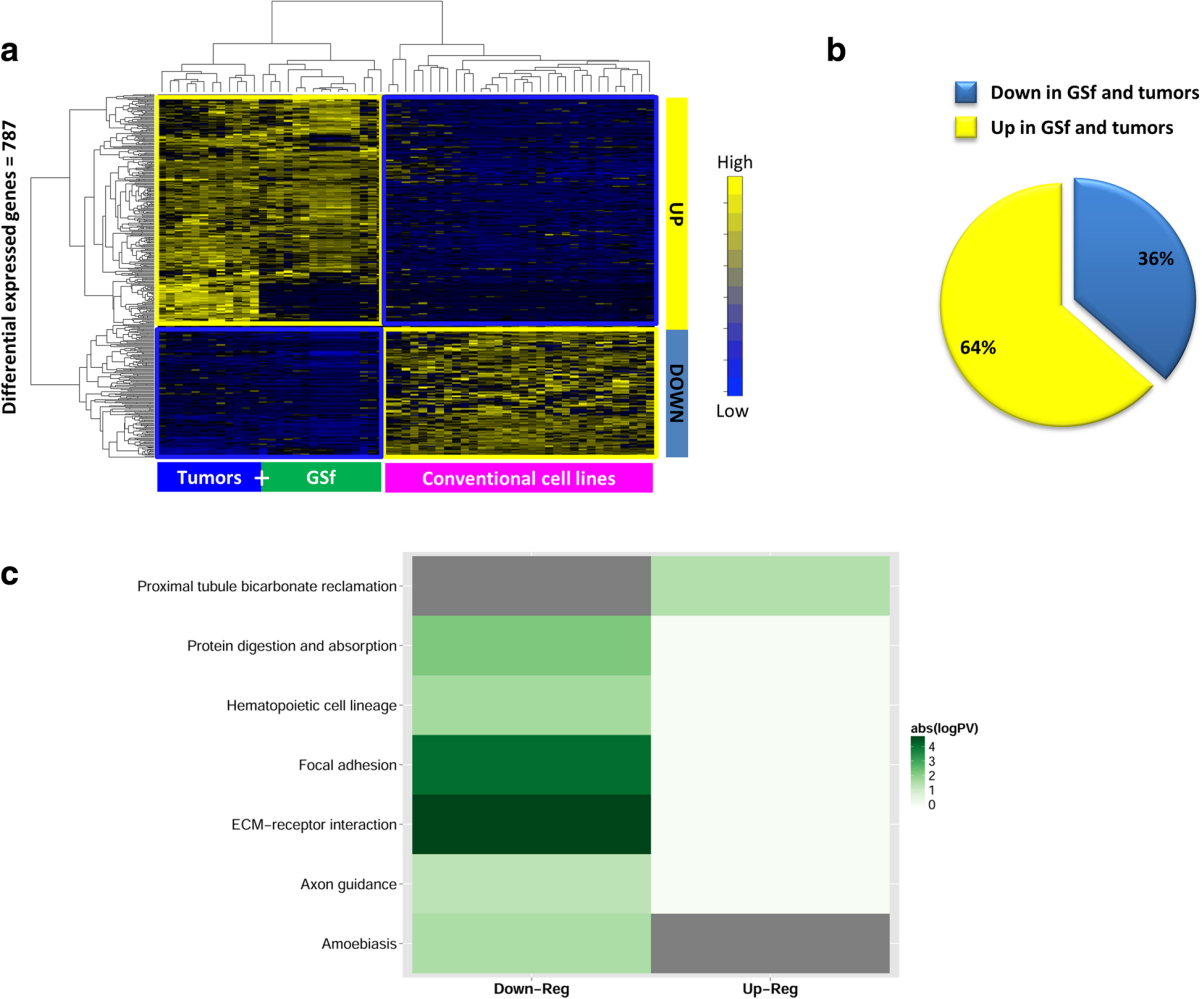
Analysis on differential expressed genes (DEGs) for Schulte et al. GBM dataset [25]. **a** Dendrogram and heat map for DEGs. The expression profiles of DEGs are clustered according to rows (genes) and columns (samples) in the GBM data matrix by using as distance metrics 1- *ρ*, where *ρ* is the Pearson correlation. Heatmap colors represent different expression levels (z-score normalized) that increase from blue to yellow. **b** Percentages of DEGs that result up-regulated and down-regulated in glioblastoma full stem-like phenotype (GSf) cell lines and primary tumors. **c** Enrichment analysis in KEGG pathways (www.genome.jp/kegg/pathway.html) for DEGs that are up-regulated and down-regulated in GSf cell lines and primary tumors. The up-regulated and down-regulated genes are considered as separate groups. The heatmap reports the absolute value increasing from white to dark green of log10 of corrected *p*-value of functional categories significantly enriched (adjusted *p*-value < 0.05) in at least one of two groups. Grey color indicates that no gene is present

By analyzing Schulte et al. dataset [25], SWIM identified 787 genes showing significant differential expression between glioblastoma full stem-like phenotype (GSf) cell lines together with tumors and conventional glioma cell lines (Fig. 3a, Additional file 1). Among them, 500 (64%) were up-regulated and only 287 (36%) were down-regulated in GSf cell lines and tumors during the stemness-differentiation transition (Fig. 3b). To provide an overview of the biological functions associated to differentially expressed genes (DEGs), we used FIDEA bioinformatics tool [36]. KEGG pathway analysis revealed that the most significantly over-represented (adjusted *p*-value < 0.05) pathways among down-regulated transcripts were “ECM-receptor interaction“ and “focal adhesion”, while the larger list of up-regulated transcripts was not found significantly enriched in relevant cancer-related pathways (Fig. 3c).

**Fig. 4:**
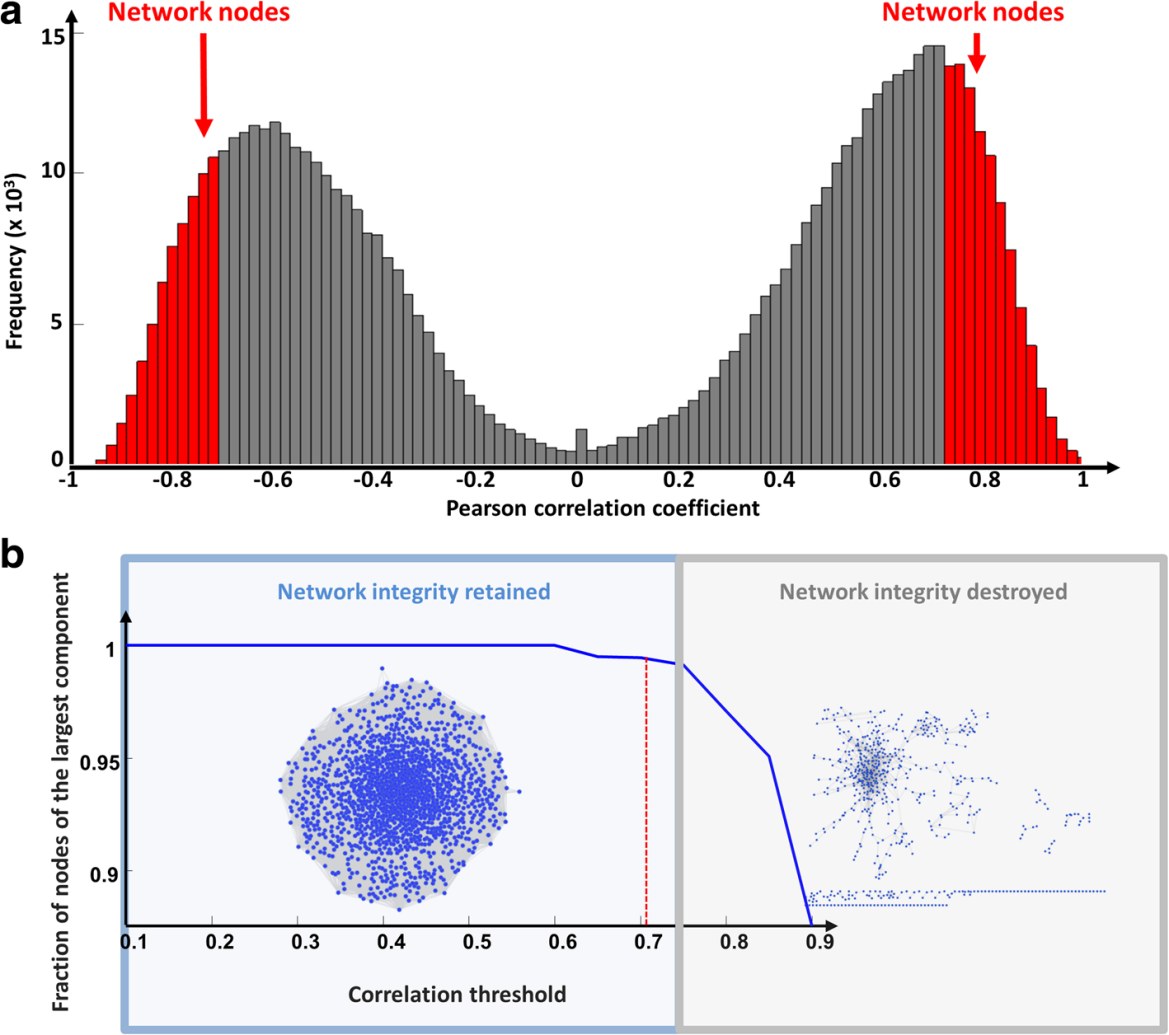
Correlation network for Schulte et al. GBM dataset [25]. (**a**) Distribution of the Person correlation coefficient between the expression profiles of all pairs of genes in the GBM network. Red (grey) regions correspond to the highly (poorly) correlated pairs. Only highly correlated pairs (red regions) will be used to build the correlation network. (**b**) Connectivity of the GBM correlation network. The x-axis represents the Pearson correlation threshold varying in the chosen range, while the y-axis represents the fraction of nodes populating the largest component. The dashed red lines correspond to the selected threshold. Note that y=1 means that all nodes fall in the largest component and thus the network is fully connected; otherwise more components exist

In order to identify potential master regulators of stemness-differentiation transition, SWIM generated a correlation network of DEGs using as distance metric the Pearson correlation coefficient between any two pairs of transcripts. Of note, the Pearson correlation distribution of all RNA profile pairs revealed a clear bimodal profile (Fig. 4a). To build the network, a Pearson correlation threshold of 0.71 was selected. It should reflect a right balance between a manageable and full connected network (see step 3 of SWIM software subsection of Methods). The co-expression network comprised 732 nodes and 75209 edges (Additional file 2 and Fig. 4b).

SWIM next searched for specific topological properties of the correlation network using the date/party/fight-club hub classification system, which we previously defined [23, 24], based on the Average Pearson Correlation Coefficients (APCCs) between the expression profiles of each hub and its nearest neighbors. The extent to which hubs are co-expressed with their interaction partners leads to three classifications with characteristic topological properties: date hubs (low positive APCC), party hubs (high positive APCC), and fight-club hubs (negative APCC). Date hubs have a coordinating role within the network, whereas party hubs act as local hubs [32]. Likewise date hubs, fight-club hubs are supposed to connect different biological processes, thus acting as global hubs, but differently from them they display an opposite transcriptional pattern with respect to their interaction partners: if they are induced, their interaction partners are repressed, and viceversa.

**Fig. 5:**
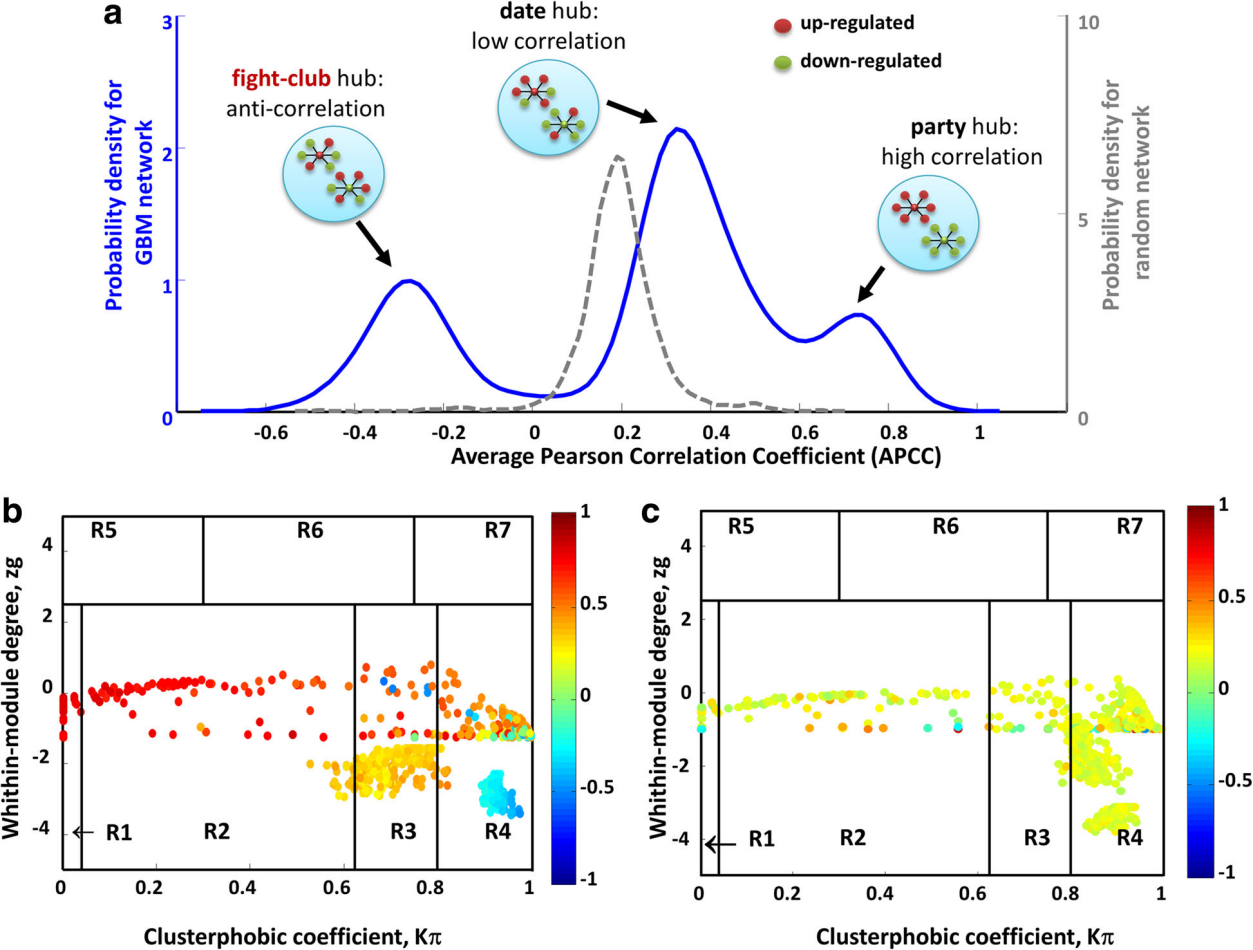
Switch genes in GBM correlation network for Schulte et al. GBM dataset [25]. **a** Probability distribution of APCC for hubs identified in the correlation network built from the glioblastoma expression dataset (blue solid line) and in its randomized counterpart obtained by shuffling the edges but preserving the degree of each node (grey dashed line). **b**-**c** Heat cartography maps where dots correspond to nodes in the GBM and random correlation network, respectively. Nodes are distributed across seven regions (R1 to R7) according to their clusterphobic coefficient *K*_*π*_ (x-axis), that is a measure the fear of each node to be confined in its own cluster, and to their within-module degree *z*_*g*_ (y-axis). Each node is colored according to its APCC value

SWIM identified 147 party hubs, 371 date hubs, and 175 fight-club hubs in the glioblastoma dataset (Additional file 3). The date/party/fight-club hub trichotomy is mirrored by the trimodal distribution of APCCs (Fig. 5a) that is emerging as distinctive feature of the complex biological correlation networks [23, 24]. In order to show that this trimodal behavior of the APCCs was not obtained by chance, we calculated the distribution of APCCs in a randomized network generated by keeping node labels constant while shuffling their edges but preserving the degree of each node. The resulting distribution was unimodal with a peak equivalent to a very low positive APCC value of ∼ 0.2 (Fig. 5a). This positive value reflects the predominance of positive compared with negative correlations in the network.

### Heat cartography in glioblastoma reveals switch genes as network bottlenecks

SWIM next searched for the communities within the glioblastoma correlation network using k-means clustering algorithm (see step 4 of SWIM software subsection of Methods), which led to the identification of three clusters or modules (Additional file 4). The intramodule and intermodule connections were exploited by SWIM in order to assign topological roles to each node [23] based on the computation of two parameters for each node: the clusterphobic coefficient *K*_*π*_, which measures the “fear” of each node of being confined in a cluster in analogy with the claustrophobic disorder, and the global within-module degree *z*_*g*_, which measures how “well-connected” each node is to other nodes in its own community. In particular, high *z*_*g*_ values correspond to nodes that are hubs within their module (local hubs), while high values of *K*_*π*_ identify nodes that interact mainly outside their community (see step 5 of SWIM software subsection of Methods). The values of these two parameters allow to define the heat cartography map for the glioblastoma dataset, where party, date, and fight-club hubs were easily identified by red, orange, and blue coloring, respectively (Fig. 5b). Fight-club hubs, acting as negative regulators, mainly fall in the so-called R4 region of the heat cartography map that is characterized by high values of the clusterphobic coefficient and by a strong inclination of nodes to interact mostly outside their own community. This subset of fight-club hubs lying in the region R4 was called *switch genes* (see step 6 of SWIM software subsection of Methods).

SWIM identified 171 switch genes out of 175 fight-club hubs in the glioblastoma dataset (Additional file 5). By drawing the heat cartography map for the nodes of the glioblastoma randomized network, we observed a predominance of low positive correlation and an absolute absence of switch genes (Fig. 5c). The parameters used for running SWIM are listed in Table 1.

**Table 1:** Parameters’ summary used by SWIM for Schulte et al. GBM dataset [25]

Parameter	Value	Number
FC-threshold	1.5	–
FDR-threshold	0.05	–
*ρ*-threshold	0.71	–
k-mean clusters	–	3
DEGs	–	787
Correlation network nodes	–	732
Correlation network edges	–	75209
Switch	–	171

Parameters column refers to: Fold-Change (FC), False Discovery Rate (FDR), Pearson correlation (*ρ*) thresholds, clusters set for k-means algorithm (k-means clusters), Differentially Expressed Genes (DEGs), nodes and edges of correlation network, and switch genes (switch)

### Characterization of switch genes

**Fig. 6:**
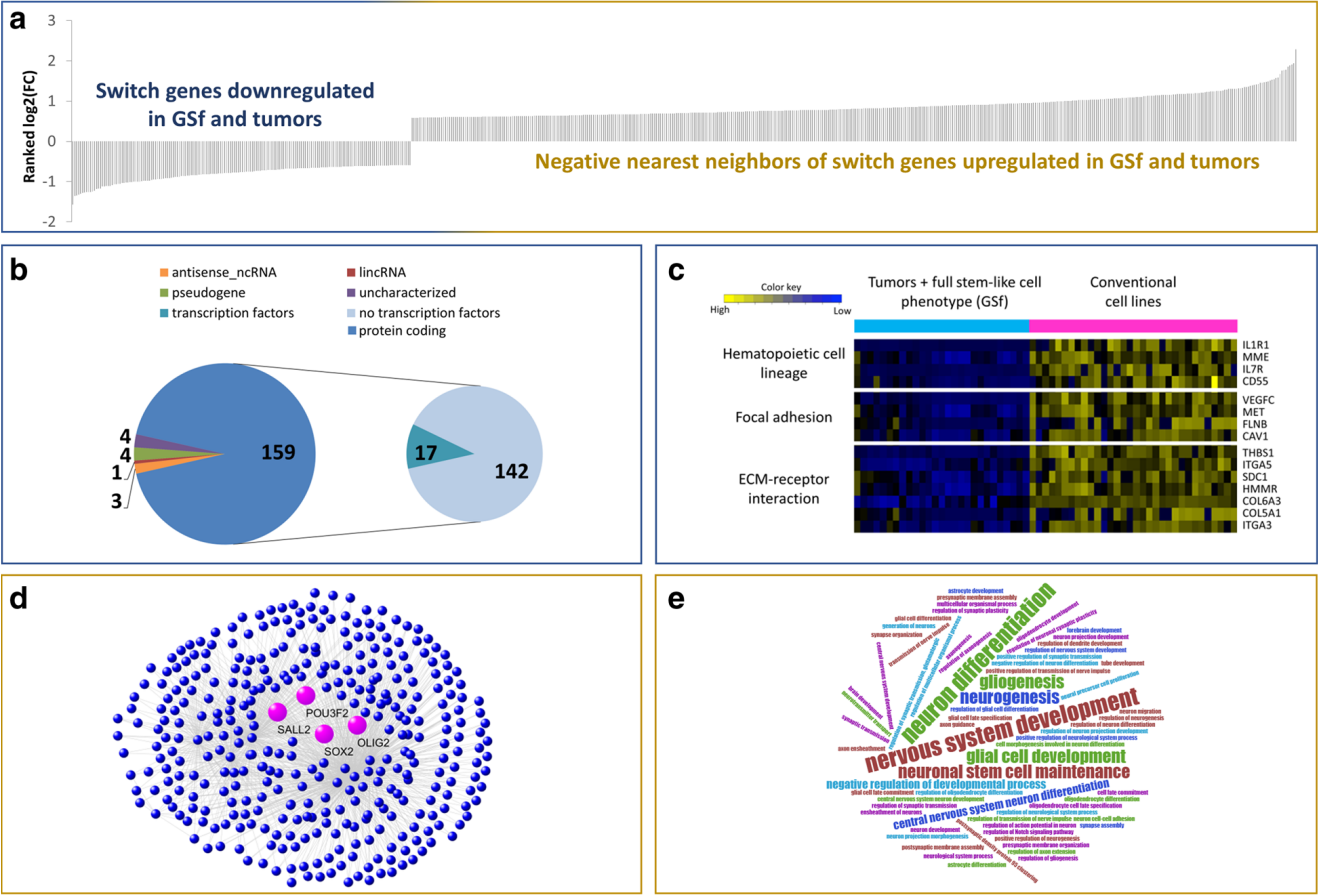
Switch genes and their negative nearest neighbors for Schulte et al. GBM dataset [25]. **a** Bar plots representing the sorted logarithmic fold-change (GSf and tumors versus conventional cell lines) for switch genes (left) and their negative nearest neighbors (right). **b** Pie charts representing the classification of switch genes according with their molecular type. **c** Heatmap representing the z-score normalized expression level of switch genes grouped according their enriched KEGG pathways. **d** Network reporting the negative nearest neighbors of the switch genes, where the core set of the well-known stemness genes is highlighted in magenta. **e** Word-cloud representing the biological processes (GO terms) over-represented in the list of the negative nearest neighbors

The list of 171 switch genes encompasses 159 protein-coding, 8 long non-coding (i.e. 4 pseudogenes, 3 antisense genes and 1 lincRNA) and 4 transcripts not characterized yet (Additional file 6 and Fig. 6b). We found 17 transcription factors (TFs) among the 159 protein-coding switch genes, including FOSL1, SNAI2, TWIST1, and WNT5A that were resulted annotated for nervous system related processes (Additional file 6). Interestingly, all switch genes were down-regulated in GSf cell lines and tumors (Additional file 5 and Fig. 6a) and enriched in the cell communication pathways “ECM-receptor interaction” and “focal adhesion” (Fig. 6c). Cell-cell adhesion is well-known to be a fundamental process for tissue architecture and morphogenesis [44] and its alteration can disrupt important cellular processes and lead to a variety of diseases, including cancer [45]. The deep-rooted evidences of the importance of cell-cell adhesion in cancer and its relation with switch genes, strongly support our hypothesis that switch genes repression may contribute to tumor invasiveness.

**Fig. 7:**
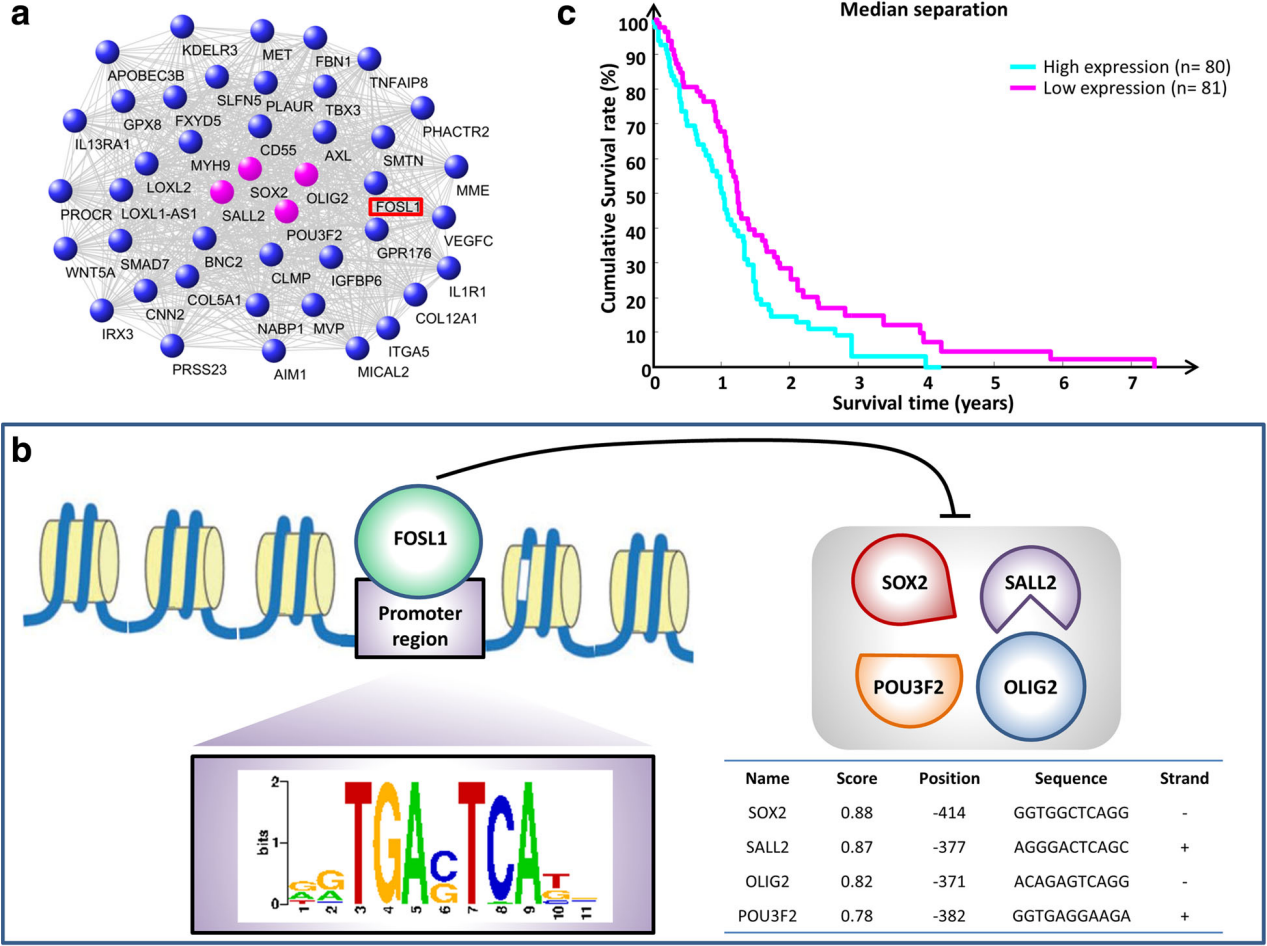
FOSL1 the most promising switch gene as therapeutic target. **a** FOSL1 belongs to the network of 41 switch genes that have, among their negative nearest neighbors, the core set of neurodevelopmental TFs (OLIG2, POU3F2, SALL2, SOX2). **c** FOSL1 results as an unfavorable prognostic marker with a log-rank test *p*-value equal to 0.019 and an adjusted *p*-value for multiple testing (Benjamini-Hochberg correction) equal to 0.3. The survival analysis was performed by using Kaplan-Meier method based on the gene expression and clinical data of 161 unique GBM patients provided by TCGA. Patients were split according to a median separation into two groups (i.e. low- and high-risk groups refer to patients with expression levels lower than or greater than the 50^*th*^ percentile of the distribution of FOSL1 expression values). **b** FOSL1 acts as putative repressor TF of the core set of neurodevelopmental TFs (OLIG2, POU3F2, SALL2, SOX2) in GBM cells. The panel depicts a schematic representation of FOSL1 consensus binding motif according to JASPAR

We found that switch genes inhibition strongly correlated with the activation in GSf lines and tumors of genes significantly enriched in processes related to the development and differentiation of the glia and neuronal cells, such as the four core of OLIG2, POU3F2, SALL2, and SOX2 (Fig. 6d-e). The latters represent four master neurodevelopmental TFs that were recently shown to be sufficient to fully reprogram differentiated glioblastoma cells into stem-like cells [12]. This finding is consistent with the existence of one master regulator among switch genes. It could control the stem-like phenotype of GBM cells simultaneosly acting as repressor of the four core TFs, thus causing the induction of differentiation of cancer stem cells, which severely halt cancer growth and invasion. Pursuing this hypothesis, we searched for switch genes that simultaneously anti-correlated with the four core TFs (Fig. 7a) and found a list of 41 switch genes (Additional file 7) that includes: WNT5A whose activation can drive GBM stem-like cell differentiation [46]; PLAUR that plays a key role in glioma cell migration and invasion acting mainly on integrins, a family of cell adhesion molecules [47]; and the FOS like transcription factor FOSL1 that appears to be expressed in various cancer tissues and associated to glioblastoma aggressiveness, invasion, and metastasis [48, 49].

Next, we investigated possible co-regulation of the four core TFs by using Pscan [37], that evaluates enrichment of known binding motifs in promoter regions, employing the JASPAR 2018 motif collection [38]. Significant enrichment was found for FOSL1 that, thus, resulted as a putative transcription factor binding to four core TFs regulatory elements (Fig. 7b).

**Fig. 8:**
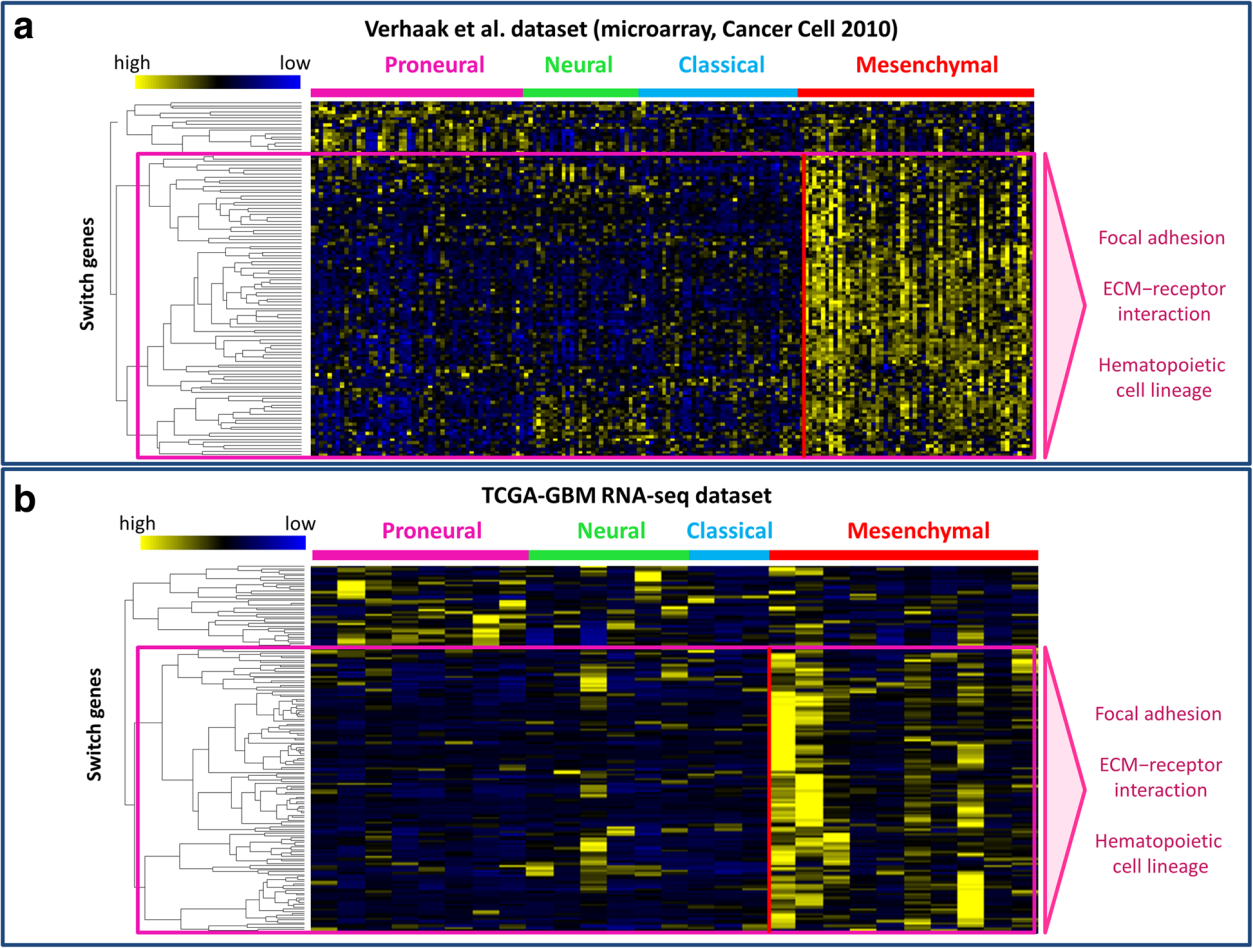
Analysis of switch genes expression of GBM subtypes. **a**-**b** Dendrograms and heat map for switch genes for microarray expression data of Verhaak et al. dataset [27] (**a**) and for GBM RNA-seq data provided by TCGA (**b**). The expression profiles of switch genes are clustered according to rows (switch genes) and columns (subtype samples) by using as distance metrics 1- *ρ*, where *ρ* is the Pearson correlation. Heatmap colors represent different expression levels (z-score normalized) that increase from blue to yellow

A recent study cataloged recurrent genomic abnormalities in glioblastoma and described a robust gene expression-based molecular classification of GBM into Proneural, Neural, Classical, and Mesenchymal subtypes by integrating multi-dimensional genomic data to establish patterns of somatic mutations and DNA copy number [27]. In order to investigate a putative connection between switch genes and the known GBM subtypes, we performed an additional analysis of their expression profiles by using both RNA-seq data downloaded from TCGA and microarray data available from this study [27]. We found connection between the four subtypes and the 171 switch genes in both datasets. Performing a hierarchical clustering, by using Pearson correlation distance as metrics, and displaying the results in the heatmap, we found that switch genes could be grouped in two main clusters (Fig. 8). In particular, those switch genes falling in the biggest cluster are enriched in “ECM-receptor interaction” and “focal adhesion” pathways and are mostly up-regulated in mesenchymal subtype that has been shown to have the worst prognosis [50]. This largest subset of switch genes includes FOSL1.

**Fig. 9:**
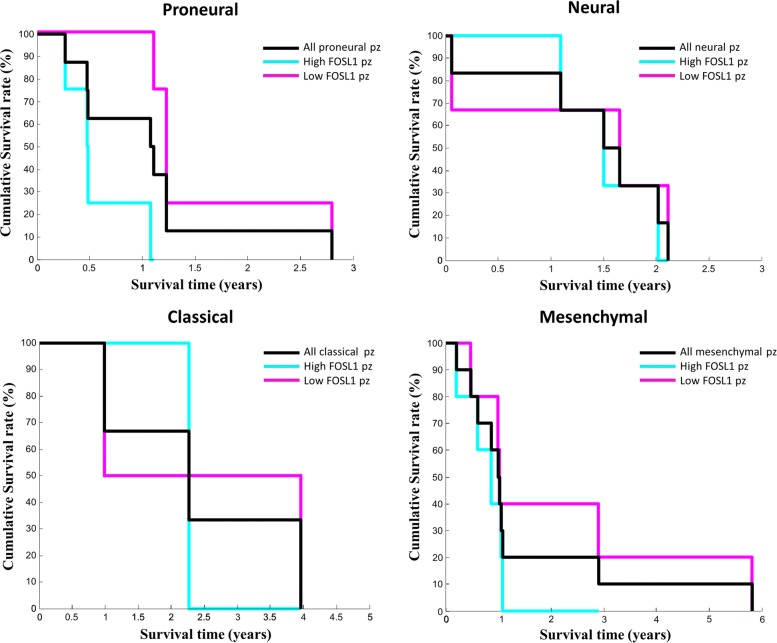
FOSL1 survival analysis by GBM subtypes. Survival analysis was performed by using Kaplan-Meier method based on the gene expression profiles of GBM RNA-seq data and clinical data provided by TCGA. For each GBM subtype (i.e. Proneural, Neural, Classical, Mensenchymal), patients are split according to a median separation into two groups: low- (magenta curve) and high-expression group (cyan curve) referring to patients with expression levels lower than or greater than the 50^*th*^ percentile of the distribution of FOSL1 expression values. Finally, we compare these results, for each subtype, with the survival curves obtained considering all patients of each subtype without the two-groups separation (black curve)

Considering all these clues that point towards FOSL1 as a possible master regulator of the four core TFs, we sought its clinical relevance by investigating its prognostic value through Kaplan-Meier survival analysis. To perform this analysis we considered the whole list of 171 switch genes and, taking advantage from the comprehensive atlas of human cancers TCGA, we correlated their expression profiles with patients survival in the GBM specific case. For each switch gene, patients were stratified into low and high risk groups through a Kaplan-Meier analysis and the statistical difference (i.e. log-rank test *p*-values) in their survivals was calculated (Additional file 8). A *p*-value adjustment for multiple testing was performed by using the Benjamini-Hochberg (FDR) procedure [28]. Unfortunately, after multiple-testing correction no switch gene were found statistically significant. However, it’s worth to note that FOSL1 appears among the top ten switch genes with the smallest *p*-value. Finally, we performed the same survival analysis also considering only patients falling in the known GBM subtypes. Overall, patients with a high FOSL1 expression show an unfavorable outcome, even if not statistically significant, both considering all 161 GBM patients and the subtype-specific patients (Figs. 7c, 9).

Recently, the expression of FOSL1 has been linked to focal adhesion closing thus the circle with the the results of the functional enrichment analysis of the switch genes that reported “ECM-receptor interaction” and “focal adhesion” as the most over-represented pathways. It has been suggested that, in a mouse model of embryonic development in vitro, FOSL1 functions as a modulator of the level of key molecules on endothelial cell surface. It can function as either an activator or a repressor, depending on the gene-context, controlling in this way the delicate equilibrium between adhesion and migration in sprouting angiogenesis [49].

Taken together these findings thrust FOSL1 into the spotlights as the most promising candidate among switch genes as novel therapeutic target for treating human glioma.

### microRNAs regulating switch genes

In order to elucidate the cascade of events underlying the maintenance of the glioblastoma stem-like cells identity, we surveyed regulatory activity of miRNAs on switch genes as computationally predicted by TargetScan [39] and experimentally validated by miRTarBase [40].

**Fig. 10:**
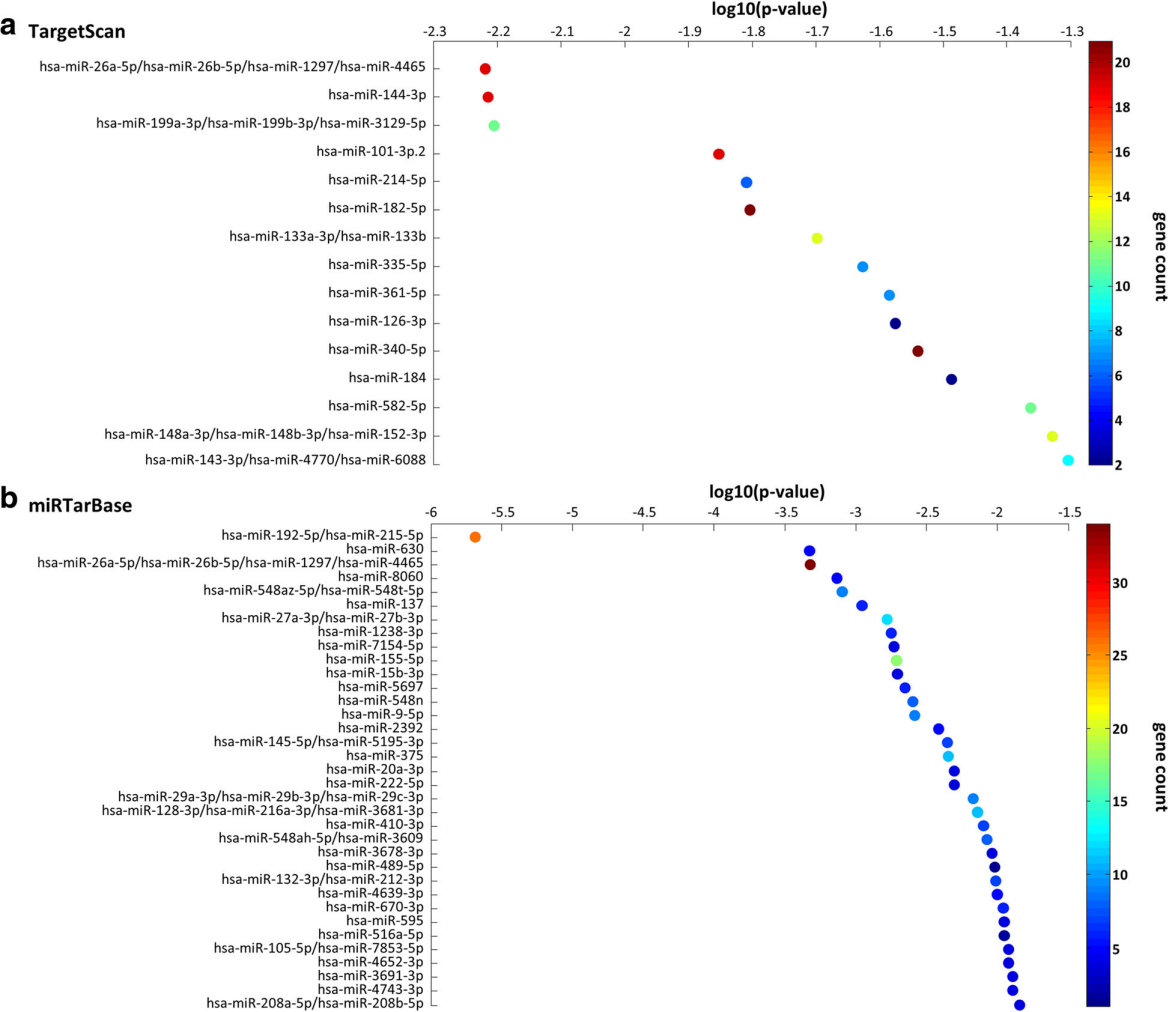
miRNA target enrichment analysis of switch genes (**a**)-(**b**) The figure shows the miRNA families found to be significantly enriched (*p*-value < 0.01) in the lists of switch genes, based on miRNA-target interaction that are computationally predicted by TargetScan (panel **a**) or experimentally validated by miRTarBase (panel **b**). The y-axis reports the most significantly over-represented miRNA families, while the x-axis reports the log10 of the *p*-value. The color code reflects the number of switch genes that are targets of each corresponding miRNA family

For each miRNA predicted to target one or more switch genes, we performed an enrichment analysis to evaluate the statistical significance (*p*-value) of the over-representation of its targets in the list of switch genes. The list of miRNAs was then sorted by increasing *p*-value (Additional file 9). Among the top-ranked miRNAs we found (Fig. 10a): the members of the miR-26 family (i.e. hsa-miR-26a-5p/hsa-miR-26b-5p/hsa-miR-1297/hsa-miR-4465), where miR-26a appears to be over-expressed in high-grade glioma and facilitates gliomagenesis in vivo [51] and miR-26b plays an important role to inhibit the proliferation and invasion of glioblastoma cells [52]; miR-144-3p recently proposed as n factor for GBM patients [53]; miR-101-3p, whose over-expression correlates with significant inhibition of in vitro proliferation and migration of glioma cells, and in vivo growth of established tumors [54]; miR-182-5p recently proposed as a prognostic marker for glioma progression and patient survival [55]; miR-340-5p recently proposed as a glioma killer and potential prognosis biomarker and therapeutic target for GBM [56]; miR-582-5p recently proposed to positively influence glioblastoma survival and promote human glioblastoma stem-cell survival [57]; the miRNA-148/-152 family (i.e. miR-148a-3p/hsa-miR-148b-3p/hsa-miR-152-3p), where miR-148a appears to be as a negative risk factor in glioblastoma and its up-regulation could accelerate the malignant process being negatively correlated with the survival rate [58].

We found that all these top-ranked miRNAs target those switch genes that are involved in “ECM-receptor interaction” and “focal adhesion” pathways - such as COL5A1, COL6A3, FLNB, ITGA5, MET, THBS1, SDC1, VEGFC - suggesting a further layer of regulation given by miRNAs that could inhibit the “ECM-receptor interaction” and “focal adhesion” pathways by directly targeting switch genes involved in them, and thus promoting cancer invasion and migration.

Similar to the above, we performed the same enrichment analysis for the experimentally validated miRNA-target interaction and once again the miR-26 family and the miR-144-3p appear as the top-ranked miRNAs (Fig. 10b).

### Topological relevance of fight-club hubs and switch genes in glioblastoma correlation networks

**Fig. 11:**
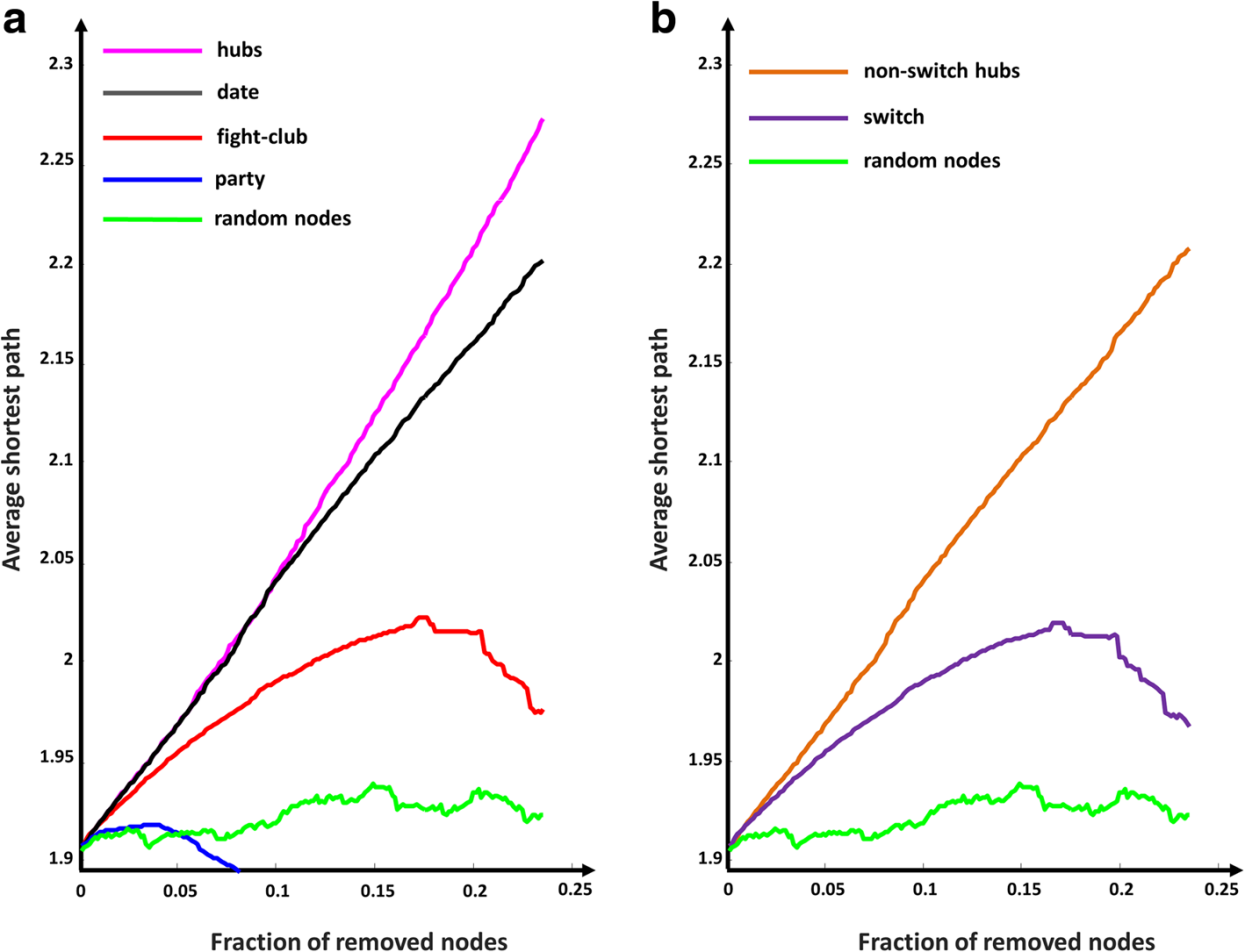
Robustness for GBM correlation network. **a**-**b** For each class of hubs, nodes are sorted by decreasing degree and the first 171 (i.e. the number of switch) sorted nodes are selected to be removed. Then, the cumulative node deletion is computed by class (i.e. total hubs, party hubs, date hubs, fight-club hubs, switch genes, and randomly chosen nodes). The x-axis represents the cumulative fraction of removed nodes with respect to the total number of network nodes that is 732, while the y-axis represents the average shortest path. Each curve corresponds to the variation of the average shortest path of the GBM correlation network as function of the removal of nodes specified by the color of each curve

In order to test the topological relevance of nodes in the GBM correlation network with respect to the overall network connectivity, SWIM evaluated the effect of their random/targeted removal on the average shortest path (see step 6 of SWIM software subsection of Methods). In particular, SWIM investigated whether fight-club, date, and party hubs as well as switch genes have distinct topological properties by evaluating the effects produced on the glioblastoma correlation networks upon their deletion (Fig. 11). Strikingly, the removal of the first 40% of fight-club hubs (i.e. the top-seventy in the ranked list of fight-club hubs sorted by decreasing degree), which corresponds to only 10% of the total nodes, produces a drastic increase of the average shortest path presumably indicating a very rapid disintegration of the network into multiple components (Fig. 11a). This beahvior is very similar to the effect caused by the deletion of the same percentage of date hubs that are known to be higher-level connectors between groups [32, 59]. On the contrary, the random removal of nodes does not effect the integrity of the network letting almost unchanged the average shortest path.

Evaluating the contribute of switch genes to the robustness of the network, we found the same behavior observed on the deletion of fight-club hubs (Fig. 11b) and the same drastic effect upon removal the first 40% of switch genes, as expected because 98% of them are switch genes. This crucial subset of switch genes encompasses FLNB, ITGA3, MET, THBS1, VEGFC, thus resulting enriched in “ECM-receptor interaction” and “focal adhesion” pathways, and also FOSL1, whose function is related to these pathways and which we acclaimed as the most promising GBM switch gene.

**Fig. 12:**
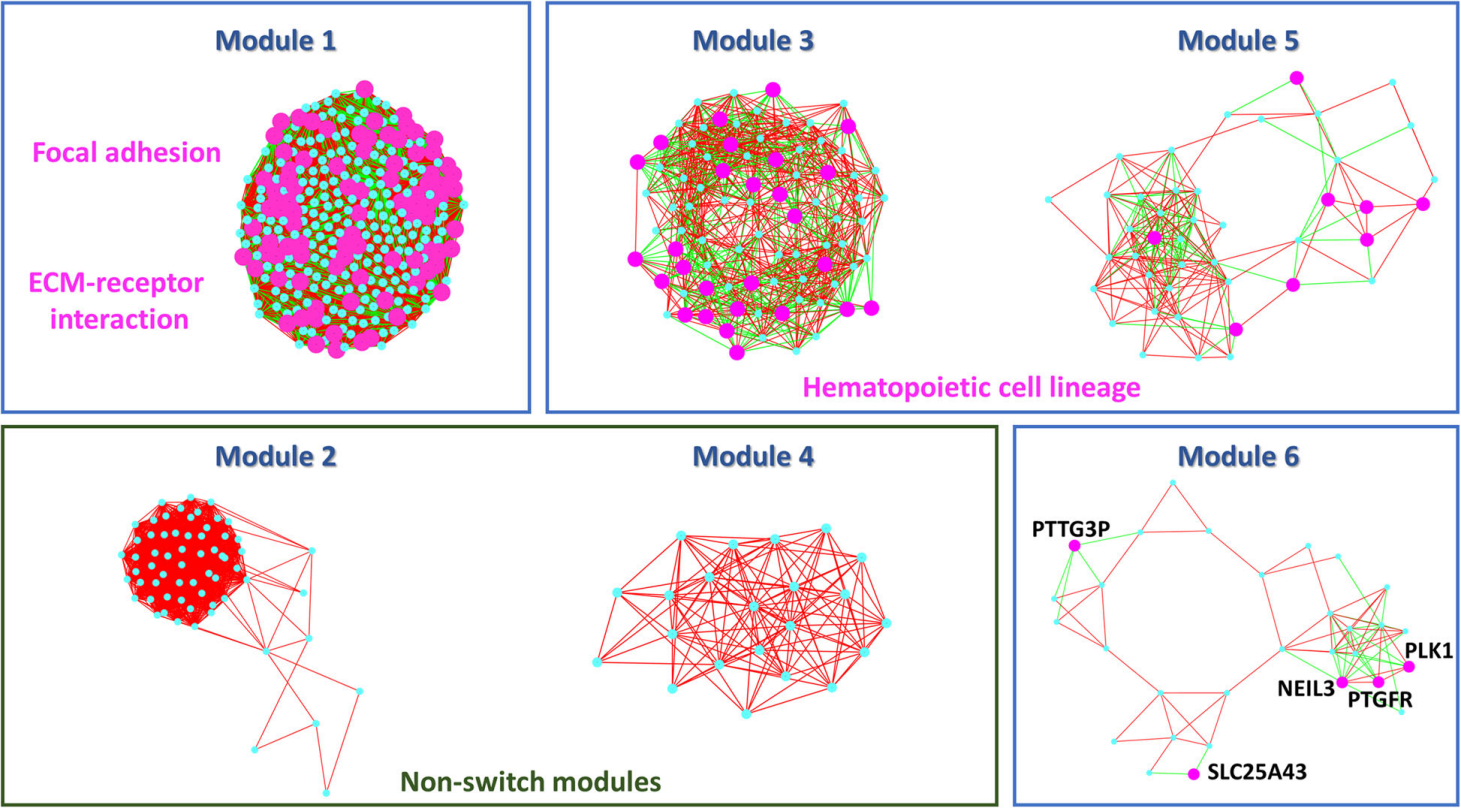
Modules in GBM correlation network. Modules (i.e. locally dense subgraphs) of GBM correlation network obtained by using the Cytoscape plugin MCODE [42] are depicted and ranked from the most densely connected subgraph to the less one. Switch genes are highlighted as magenta bigger circles in each module and the corresponding enriched KEGG pathways are reported

To further strengthen the topological relevance of switch genes, we divided the GBM network in densely connected subgraphs and we found that the above-mentioned crucial subset of switch genes falls in the most locally dense module (Fig. 12). All these findings point to a crucial role of “ECM-receptor interaction” and “focal adhesion” pathways in the GBM regulatory network through the switch genes directly or indirectly associated to them.

## Discussion

Nowadays, the GBM research scene is dominated by trying to discover novel therapeutic and prognostic markers promoting the differentiation of cancer stem-like cells, which severely halts cancer growth and might affect the therapeutic outcome. Although the adjusted *p*-values for multiple testing didn’t reveal any statistically significant association (*p*-value < 0.05) of switch genes with patient survival, the transcription factor FOSL1 fulfills very interesting features that make it eligible as new potential therapeutic target. In particular: it was found to act as repressor transcription factor [49]; it resulted down-regulated in stem-like cells; it resulted highly negatively correlated with the 4-core TFs that were resulted all up-regulated in stem-like cells; the promoter regions of the 4-core TFs were found to harbor a consensus binding motif for FOSL1. Taken together these considerations prompt us to bet on FOSL1, which can promote the differentiation process of GBM stem-like cells by repressing the 4-core TFs. This should allow for anticipation of care as well as the reduction of the social impact of diseases and the restraint of health costs.

## Conclusion

Although our study can be considered as a starting point, and further functional and clinical investigations are needed, the switch gene signatures and their nearest neighbor genes can improve our knowledge of the cellular events that are crucial for carcinogenesis and they also reveal many potential prognostic and novel therapeutic targets that have so far not been linked to glioblastoma. Thus, using SWIM could provide important clues that will stimulate research activities into the causes of this terrible disease thus supporting the planning of healthcare services such as clinical trials and disease prevention.

## Additional files

Additional file 1 Table. GBM differentially expressed genes for Schulte et al. dataset [25]. The table reports the list of the statistically significant (adjusted *p*-value <0.05) DEGs in Official Gene Symbols identified by SWIM in human GBM dataset analyzed in the present study; the *p*-values resulted from an unpaired two-tailed Student t-test; the adjusted *p*-values; the fold-change values; the DEGs expression direction (up/down in GSf and tumors); the percentage of the up- and down-regulated DEGs in a pie chart visualization. (XLSX 76 kb)

Additional file 2 Table. GBM correlation network for Schulte et al. dataset [25]. The table reports the co-expression network of DEGs identified by SWIM in GBM dataset analyzed in the present study. Source nodes, target nodes and their interaction (Pearson correlation) are listed. (XLSX 1930 kb)

Additional file 3 Table. Roles of nodes in GBM correlation network for Schulte et al. dataset [25]. The table contains the following columns. *nodeName*: the names of the nodes of the correlation network (Official Gene Symbol); *hub*: it specifies if the nodes of the heat cartography map are (or are not) local hubs within their community; *Region*: it specifies the region of the heat cartography map to which each node belongs; *Type*: it specifies for each node of the heat cartography map its universal role; *Kp*: values of the clusterphobic coefficient parameter *K*_*π*_; *zg*: values of the within-module degree parameter *z*_*g*_; *APCC*: the average of the Pearson correlation coefficients between the expression profiles of a node and those of its interaction partners in the heat cartography map; *Degree*: the degree for each node in the heat cartography map; *Date-Party*: if the node of the heat cartography map is either a date or party or fight-club hub or if it is not a local hub in its community. (XLSX 64 kb)

Additional file 4 Figure. Scree plot for the choice of the k-means number of clusters. The x-axis represents the number of clusters, while the y-axis represents the sum of the squared error (SSE). The SSE is computed as sum of the distance of each object to its closest centroid and the number of clusters is chosen on the basis of the elbow position. (TIF 124 kb)

Additional file 5 Table. GBM switch genes identified by SWIM for Schulte et al. dataset [25]. The table reports: the list of the switch genes in Official Gene symbol; the adjusted *p*-values; the fold-change values; their expression direction (up/down in GSf and tumors). (XLSX 16 kb)

Additional file 6 Table. Switch genes functional annotations and molecular type. The table is composed of three sheets reporting: the list of the switch genes in Official Gene symbol together with their GO term annotations provided by BioMart [60], whenever available; an indication of their molecular type (protein coding, non-coding or miRNA) obtained from Ensembl annotations of the human genome (GHCR38 version), whenever available; the list of switch genes characterized by a transcription factor activity. The TFs annotated for nervous system related processes are highlighted in yellow. (XLSX 87 kb)

Additional file 7 Table. Switch genes negatively interacting with four core TFs. The table reports the list of switch genes that are simultaneously anti-correlated with OLIG2, POU3F2, SALL2, and SOX2. (XLSX 7 kb)

Additional file 8 Table. Kaplan-Meier survival analysis. The table reports the list of the switch genes, the *p*-values resulted from log-rank test, and the adjusted *p*-values (FDR) for multiple testing. The list is sorted by increasing *p*-values. The file reports the 161 switch genes out of 171, whose expression data were available on TCGA Data Portal Release 10.0 (December 2017). (XLS 10 kb)

Additional file 9 Table. Enrichment analysis of miRNA-target interactions among switch genes. The table reports miRNA families found to be enriched in the lists of switch genes, based on the miRNA-target interactions computationally predicted by TargetScan (sheet 1) and experimentally validated by miRTarBase (sheet 2). The miRNA families are sorted according to the increasing *p*-values. (XLSX 159 kb)

## Abbreviations

APCC: Average pearson correlation coefficient
DEGs: Differentially expressed genes
ECM: Extracellular matrix
FC: Fold-change
FDR: False discovery rate
GBM: Glioblastoma multiforme
GEO: Gene expression omnibus
GO: Gene ontology
GS: Glioblastoma stem-like
GSf: Glioblastoma full stem-like phenotype
GUI: Graphical user interphase
IQR: Inter quartile range
miRNA: microRNA
PPI: Protein-protein interaction
SSE: Sum of the squared error
SWIM: SWItchMiner
TCGA: The cancer genome atlas
TFs: Transcription factors
WHO: World health organization

## References

[1] Jansen M, Yip S, Louis DN (2010). Molecular pathology in adult gliomas: diagnostic, prognostic, and predictive markers. Lancet Neurol.

[2] Young RM, Jamshidi A, Davis G, Sherman JH (2015). Current trends in the surgical management and treatment of adult glioblastoma. Ann Transl Med.

[3] Ostrom QT, Gittleman H, Fulop J, Liu M, Blanda R, Kromer C, Wolinsky Y, Kruchko C, Barnholtz-Sloan JS (2015). Cbtrus statistical report: Primary brain and central nervous system tumors diagnosed in the united states in 2008-2012. Neuro-Oncol.

[4] Grossman SA, Ye X, Piantadosi S, Desideri S, Nabors LB, Rosenfeld M, Fisher J, Consortium NC (2010). Survival of patients with newly diagnosed glioblastoma treated with radiation and temozolomide in research studies in the united states. Clin Cancer Res.

[5] Mizoe J-E, Tsujii H, Hasegawa A, Yanagi T, Takagi R, Kamada T, Tsuji H, Takakura K, of the Central Nervous System Tumor Working Group OC, et al. Phase i/ii clinical trial of carbon ion radiotherapy for malignant gliomas: combined x-ray radiotherapy, chemotherapy, and carbon ion radiotherapy. Int J Radiat Oncol* Biol* Phys. 2007; 69(2):390–6. 10.1016/j.ijrobp.2007.03.003 17459607

[6] Sathornsumetee S, Rich JN (2008). Designer therapies for glioblastoma multiforme. Ann NY Acad Sci.

[7] Weathers S-P, Gilbert MR (2014). Advances in treating glioblastoma. F1000Prime Rep.

[8] Weinstein JN, Collisson EA, Mills GB, Shaw KRM, Ozenberger BA, Ellrott K, Shmulevich I, Sander C, Stuart JM, Network CGAR (2013). The cancer genome atlas pan-cancer analysis project. Nat Genet.

[9] McLendon R, Friedman A, Bigner D, Van Meir EG, Brat DJ, Mastrogianakis GM, Olson JJ, Mikkelsen T, Lehman N, Aldape K (2008). Comprehensive genomic characterization defines human glioblastoma genes and core pathways. Nature.

[10] Singh SK, Hawkins C, Clarke ID, Squire JA, Bayani J, Hide T, Henkelman RM, Cusimano MD, Dirks PB (2004). Identification of human brain tumour initiating cells. Nature.

[11] Brower JV, Clark PA, Lyon W, Kuo JS (2014). Micrornas in cancer: Glioblastoma and glioblastoma cancer stem cells. Neurochem Int.

[12] Suva ML, Rheinbay E, Gillespie SM, Patel AP, Wakimoto H, Rabkin SD, Riggi N, Chi AS, Cahill DP, Nahed BV (2014). Reconstructing and reprogramming the tumor-propagating potential of glioblastoma stem-like cells. Cell.

[13] Bao S, Wu Q, McLendon RE, Hao Y, Shi Q, Hjelmeland AB, Dewhirst MW, Bigner DD, Rich JN (2006). Glioma stem cells promote radioresistance by preferential activation of the DNA damage response. Nature.

[14] Singh SK, Clarke ID, Terasaki M, Bonn VE, Hawkins C, Squire J, Dirks PB (2003). Identification of a cancer stem cell in human brain tumors. Cancer Res.

[15] Chen R, Nishimura MC, Bumbaca SM, Kharbanda S, Forrest WF, Kasman IM, Greve JM, Soriano RH, Gilmour LL, Rivers CS (2010). A hierarchy of self-renewing tumor-initiating cell types in glioblastoma. Cancer Cell.

[16] Tabatabai G, Weller M (2011). Glioblastoma stem cells. Cell Tissue Res.

[17] Guo W, Lasky JL, Wu H (2006). Cancer stem cells. Pediatr Res.

[18] Al-Hajj M, Wicha MS, Benito-Hernandez A, Morrison SJ, Clarke MF (2003). Prospective identification of tumorigenic breast cancer cells. Proc Natl Acad Sci.

[19] O’Brien CA, Pollett A, Gallinger S, Dick JE (2007). A human colon cancer cell capable of initiating tumour growth in immunodeficient mice. Nature.

[20] Lang S, Frame F, Collins A (2009). Prostate cancer stem cells. J Pathol.

[21] Li C, Heidt DG, Dalerba P, Burant CF, Zhang L, Adsay V, Wicha M, Clarke MF, Simeone DM (2007). Identification of pancreatic cancer stem cells. Cancer Res.

[22] Schmidt P, Kopecky C, Hombach A, Zigrino P, Mauch C, Abken H (2011). Eradication of melanomas by targeted elimination of a minor subset of tumor cells. Proc Natl Acad Sci.

[23] Paci P, Colombo T, Fiscon G, Gurtner A, Pavesi G, Farina L (2016). Swim: a computational tool to unveiling crucial nodes in complex biological networks. Sci Rep.

[24] Palumbo MC, Zenoni S, Fasoli M, Massonnet M, Farina L, Castiglione F, Pezzotti M, Paci P (2014). Integrated network analysis identifies fight-club nodes as a class of hubs encompassing key putative switch genes that induce major transcriptome reprogramming during grapevine development. Plant Cell.

[25] Schulte A, Günther HS, Phillips HS, Kemming D, Martens T, Kharbanda S, Soriano RH, Modrusan Z, Zapf S, Westphal M (2011). A distinct subset of glioma cell lines with stem cell-like properties reflects the transcriptional phenotype of glioblastomas and overexpresses cxcr4 as therapeutic target. Glia.

[26] Barrett T, Wilhite SE, Ledoux P, Evangelista C, Kim IF, Tomashevsky M, Marshall KA, Phillippy KH, Sherman PM, Holko M (2013). Ncbi geo: archive for functional genomics data sets—update. Nucleic Acids Res.

[27] Verhaak RG, Hoadley KA, Purdom E, Wang V, Qi Y, Wilkerson MD, Miller CR, Ding L, Golub T, Mesirov JP (2010). Integrated genomic analysis identifies clinically relevant subtypes of glioblastoma characterized by abnormalities in pdgfra, idh1, egfr, and nf1. Cancer Cell.

[28] Benjamini Y, Hochberg Y (1995). Controlling the false discovery rate: a practical and powerful approach to multiple testing. J R Statist Soc B.

[29] Hartigan JA, Wong MA (1979). Algorithm as 136: A k-means clustering algorithm. J R Stat Soc Ser C (Appl Stat).

[30] Meilă M (2006). The uniqueness of a good optimum for k-means. Proceedings of the 23rd International Conference on Machine Learning.

[31] Lisboa PJ, Etchells TA, Jarman IH, Chambers SJ (2013). Finding reproducible cluster partitions for the k-means algorithm. BMC Bioinformatics.

[32] Han J-DJ, Bertin N, Hao T, Goldberg DS, Berriz GF, Zhang LV, Dupuy D, Walhout AJ, Cusick ME, Roth FP (2004). Evidence for dynamically organized modularity in the yeast protein–protein interaction network. Nature.

[33] Guimera R, Amaral LAN (2005). Functional cartography of complex metabolic networks. Nature.

[34] Ashburner M, Ball CA, Blake JA, Botstein D, Butler H, Cherry JM, Davis AP, Dolinski K, Dwight SS, Eppig JT (2000). Gene ontology: tool for the unification of biology. Nat Genet.

[35] Kanehisa M, Sato Y, Kawashima M, Furumichi M, Tanabe M (2016). Kegg as a reference resource for gene and protein annotation. Nucleic Acids Res.

[36] D’Andrea D, Grassi L, Mazzapioda M, Tramontano A (2013). Fidea: a server for the functional interpretation of differential expression analysis. Nucleic Acids Res.

[37] Zambelli F, Pesole G, Pavesi G (2009). Pscan: finding over-represented transcription factor binding site motifs in sequences from co-regulated or co-expressed genes. Nucleic Acids Res.

[38] Khan A, Fornes O, Stigliani A, Gheorghe M, Castro-Mondragon JA, van der Lee R, Bessy A, Chèneby J, Kulkarni SR, Tan G, Baranasic D, Arenillas DJ, Sandelin A, Vandepoele K, Lenhard B, Ballester B, Wasserman WW, Parcy F, Mathelier A (2018). Jaspar 2018: update of the open-access database of transcription factor binding profiles and its web framework. Nucleic Acids Res.

[39] Agarwal V, Bell GW, Nam J-W, Bartel DP (2015). Predicting effective microrna target sites in mammalian mrnas. eLife.

[40] Chou C-H, Chang N-W, Shrestha S, Hsu S-D, Lin Y-L, Lee W-H, Yang C-D, Hong H-C, Wei T-Y, Tu S-J (2015). mirtarbase 2016: updates to the experimentally validated mirna-target interactions database. Nucleic Acids Res.

[41] Cline MS, Smoot M, Cerami E, Kuchinsky A, Landys N, Workman C, Christmas R, Avila-Campilo I, Creech M, Gross B (2007). Integration of biological networks and gene expression data using cytoscape. Nat Protoc.

[42] Bader GD, Hogue CW (2003). An automated method for finding molecular complexes in large protein interaction networks. BMC Bioinformatics.

[43] Rich JT, Neely JG, Paniello RC, Voelker CC, Nussenbaum B, Wang EW (2010). A practical guide to understanding kaplan-meier curves. Otolaryngol Head Neck Surg.

[44] Gumbiner BM (1996). Cell adhesion: the molecular basis of tissue architecture and morphogenesis. Cell.

[45] Hirohashi S, Kanai Y (2003). Cell adhesion system and human cancer morphogenesis. Cancer Sci.

[46] Hu B, Wang Q, Wang YA, Hua S, Sauvé C-EG, Ong D, Lan ZD, Chang Q, Ho YW, Monasterio MM (2016). Epigenetic activation of wnt5a drives glioblastoma stem cell differentiation and invasive growth. Cell.

[47] Veeravalli KK, Rao JS (2012). Mmp-9 and upar regulated glioma cell migration. Cell Adhes Migr.

[48] Zhang L, Liu H, Mu X, Cui J, Peng Z (2017). Dysregulation of fra1 expression by wnt/ *β*-catenin signalling promotes glioma aggressiveness through epithelial–mesenchymal transition. Biosci Rep.

[49] Galvagni F, Orlandini M, Oliviero S (2013). Role of the ap-1 transcription factor fosl1 in endothelial cells adhesion and migration. Cell Adhes Migr.

[50] Parsons DW, Jones S, Zhang X, Lin JC-H, Leary RJ, Angenendt P, Mankoo P, Carter H, Siu I-M, Gallia GL (2008). An integrated genomic analysis of human glioblastoma multiforme. Science.

[51] Huse JT, Brennan C, Hambardzumyan D, Wee B, Pena J, Rouhanifard SH, Sohn-Lee C, Le Sage C, Agami R, Tuschl T (2009). The pten-regulating microrna mir-26a is amplified in high-grade glioma and facilitates gliomagenesis in vivo. Genes Dev.

[52] Jiang Q, Liu Y, Zhang S, Li N, Sun G. Mir-26b suppresses cell proliferation and invasion by targeting cyclooxygenase 2 in human glioblastoma. Oncotarget. 2016. 10.18632/oncotarget.12706.

[53] Cheng Z, Song Y, Wang Z, Wang Y, Dong Y (2017). mir-144-3p serves as a tumor suppressor by targeting fzd7 and predicts the prognosis of human glioblastoma. Eur Rev Med Pharmacol Sci.

[54] Ma C, Zheng C, Bai E, Yang K (2016). mir-101 inhibits glioma cell invasion via the downregulation of cox-2. Oncol Lett.

[55] Jiang L, Mao P, Song L, Wu J, Huang J, Lin C, Yuan J, Qu L, Cheng S-Y, Li J (2010). mir-182 as a prognostic marker for glioma progression and patient survival. Am J Pathol.

[56] Huang D, Qiu S, Ge R, He L, Li M, Li Y, Peng Y (2015). mir-340 suppresses glioblastoma multiforme. Oncotarget.

[57] Floyd DH, Zhang Y, Dey BK, Kefas B, Breit H, Marks K, Dutta A, Herold-Mende C, Synowitz M, Glass R, Abounader R, Purow BW (2014). Novel anti-apoptotic micrornas 582-5p and 363 promote human glioblastoma stem cell survival via direct inhibition of caspase 3, caspase 9, and bim. PLoS ONE.

[58] Li Y, Deng X, Zeng X, Peng X (2016). The role of mir-148a in cancer. J Cancer.

[59] Albert R., Jeong H., Barabási A-L (2000). Error and attack tolerance of complex networks. Nature.

[60] Kinsella RJ, Kähäri A, Haider S, Zamora J, Proctor G, Spudich G, Almeida-King J, Staines D, Derwent P, Kerhornou A (2011). Ensembl biomarts: a hub for data retrieval across taxonomic space. Database.

