# Supplementary figures and images for: SWIM tool application to expression data of glioblastoma stem-like cell lines, corresponding primary tumors and conventional glioma cell lines
Source: BMC Bioinformatics. 2018 Nov 30;19(Suppl 15):436. doi: 10.1186/s12859-018-2421-x (PMC6266956; doi:10.1186/s12859-018-2421-x)

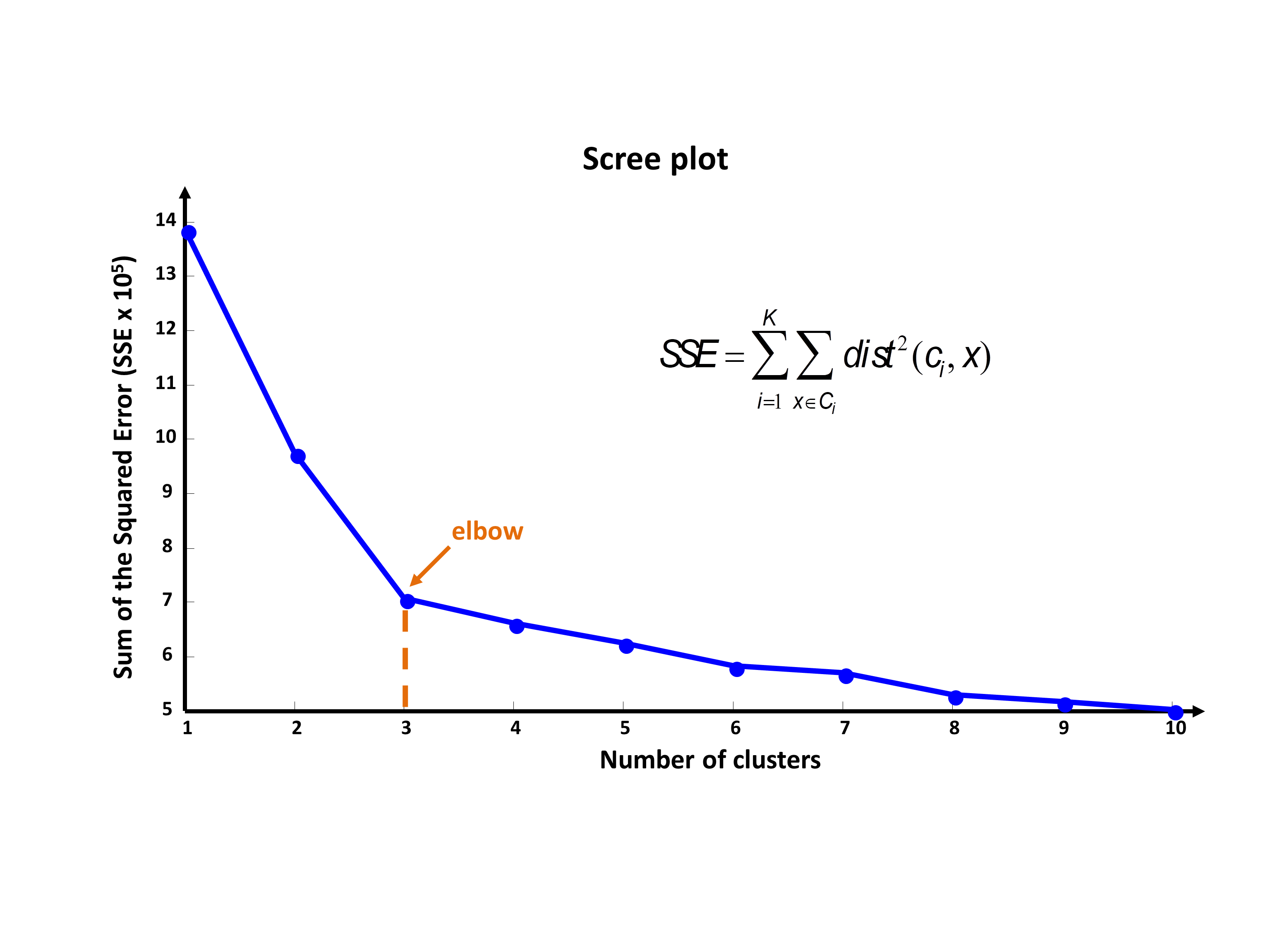

Supplement: Supplementary file 4 — Figure. Scree plot for the choice of the k-means number of clusters. The x-axis represents the number of clusters, while the y-axis represents the sum of the squared error (SSE). The SSE is computed as sum of the distance of each object to its closest centroid and the number of clusters is chosen on the basis of the elbow position. (TIF 124 kb) [file 12859_2018_2421_MOESM4_ESM.tif]
